# Supplementary material for: A Bayesian computational model reveals a failure to adapt interoceptive precision estimates across depression, anxiety, eating, and substance use disorders
Source: PLoS Comput Biol. 2020 Dec 14;16(12):e1008484. doi: 10.1371/journal.pcbi.1008484 (PMC7769623; doi:10.1371/journal.pcbi.1008484)
Supplement: S1 Text — (DOCX) [file pcbi.1008484.s001.docx]

**Supplementary Analyses:** **A Bayesian computational model reveals a failure to adapt interoceptive precision estimates across depression, anxiety, eating, and substance use disorders**

Ryan Smith^1*^, Rayus Kuplicki^1^, Justin Feinstein^1,2^, Katherine L. Forthman, Jennifer L. Stewart^1,2^, Martin P. Paulus^1,2^, Tulsa 1000 investigators, and Sahib S. Khalsa^1,2^

*Email: rsmith@laureateinstitute.org

^1^Laureate Institute for Brain Research, Tulsa, Oklahoma, United States of America

^2^Oxley College of Health Sciences, The University of Tulsa, Tulsa, Oklahoma, United States of America.

**Parameter recoverability**

To ensure that parameters were recoverable during estimation for the winning model, we generated simulated tapping data based on each possible combination of values for *IP_raw_* and *pHB* from 0.1 to 0.9 in increments of 0.1 (i.e., 81 combinations in total, where the simulated choice to tap on each trial was sampled from the posterior distribution over the heartbeat/no heartbeat states). Using the same prior means and variances as in the estimation of empirical data, we then ran identical estimation procedures and correlations to test whether parameter estimates were strongly associated with the true parameters that generated the data. For true and estimated *IP_raw_* values, we observed a correlation of *r* = 0.98 (*p* < .001). For true and estimated *pHB* values, we observed a correlation of *r* = 0.98 (*p* < .001). This justifies strong confidence in parameter recoverability.

**Group by condition interactions for anticipatory vs. reactive strategy**

Focusing only on the interoceptive conditions, and grouping participants transdiagnostically (i.e., based on the presence vs. absence of a diagnosis), a further LME revealed a main effect of medication status (*F(1,407)* = 4.10, *p* = .04), heart rate (*F(1,564)* = 11.21, *p* < .001), and group (*F(1,557)* = 6.16, *p* = .01), and a heart rate by group interaction (*F(1,562)* = 6.51, *p* = .01), but no effect of condition (*F(2,904)* = .41, *p* = .66) or group by condition interaction (*F(2,838)* = .29, *p* = .75). There was also no significant effect of any other covariate (*F*s between .02 and 1.89, *p*s between .17 and .88). The effect of medication reflected a stronger tendency to tap in a reactive manner in medicated individuals. Post-hoc contrasts showed no mean differences in AvR between groups (*p* = .43), and correlation analyses showed a non-significant relationship between AvR and heart rate (*r* = -.001, *p* = .95). Subsequent plot inspection suggested that the main effects and interaction between group and heart rate were explained by a relatively more negative relationship between AvR and heart rate in HCs than in the transdiagnostic group (*r* = -.03 and .003, respectively). An analogous LME that separated the 5 clinical groups only found an effect of medication status (*F(1,399)* = 5.19, *p* = .02), whereas all other effects observed in the earlier LME were not significant. Bayesian analyses strongly favored the null model over all other models (Bayes factors between 7.36 and > 5.25E+5 favoring the null model). See **S4 Fig** for plots of AvR values by group and condition.

**Group by condition interactions in other task-relevant variables**

To assess potential group differences in the effect of task condition on self-reported experience and physiology, we also carried analogous LME analyses assessing confidence, intensity, and difficulty, as well as heart rate (including the same list of covariates as in the analyses of IP and pHB in the main text).

*Self-reported heartbeat intensity***.** For the transdiagnostic (HCs vs all patients) LME analysis, there was a main effect of PTT (*F(1,415)* = 5.22, *p* = .02), reflecting lower felt intensity with greater PTT values. No other significant main effects or interactions were observed. The diagnosis-specific (HCs vs. 5 separate patient groups) LME analysis showed qualitatively identical results. Bayes factor analyses assessing the space of models with vs. without main effects and interactions between group and condition, found the most evidence for a model with an effect of condition and diagnosis-specific groups (Bayes factor = 1.8E+31 relative to the null model). It had very strong evidence compared to the 2^nd^-best model, which included trial and the transdiagnostic grouping (Bayes factor = 71.6). Uncorrected post-hoc contrasts suggested the effect of condition reflected greater self-reported intensity in the breath-hold condition relative to the other conditions (*p* < .001 for each) and greater intensity in the guessing than no-guessing condition (*p* = .048) when not including the covariates in the LMEs. The effect of diagnosis-specific group appeared to be explained by greater intensity in the SUD group relative to all other groups (*p*s between < .001 and .02) when not including the covariates in the LMEs.

*Self-reported confidence***.** For the transdiagnostic (HCs vs all patients) LME analysis, there was a main effect of group (*F(1,765)* = 5.24, *p* = .02) and a marginal heart rate by group interaction (*F(1,781)* = 3.90, *p* = .05), reflecting marginally higher confidence in the patient group (*p* = .096), and a relatively stronger (non-significant) positive correlation with heart rate in HCs than patients (*r* = .03 and -.01, respectively). No other significant main effects or interactions were observed. The diagnosis-specific (HCs vs. 5 separate patient groups) LME analysis showed no significant main effects or interactions. Bayes factor analyses, assessing the space of models with vs. without main effects and interactions between group and condition, found the most evidence for a model including only an effect of condition (Bayes factor = 6.3E+12 compared to the null model). There was positive evidence for this model compared to the 2^nd^-best model (with both transdiagnostic group and trial; Bayes factor = 2.9), and strong evidence compared to the 3^rd^-best model (with diagnosis-specific grouping; Bayes factor = 8.4). Uncorrected post-hoc contrasts suggested that the effect of condition in the winning model reflected greater confidence in the breath-hold condition than the other two conditions (*p* < .001) across all participants when not accounting for the covariates included in the LMEs. The effect of diagnostic group in the 2^nd^-best model appeared to correspond to a numerically greater confidence level in the transdiagnostic patient group (*p* = .17) when not accounting for the covariates included in the LMEs.

*Self-reported difficulty***.** For the transdiagnostic (HCs vs all patients) LME analysis, there was a main effect of sex (*F(1,412)* = 5.03, *p* = .03) and BMI (*F(1,408)* = 6.70, *p* = .01), reflecting greater difficulty in females and a positive relationship with BMI. No other significant main effects or interactions were observed. The diagnosis-specific (HCs vs. 5 separate patient groups) LME analysis showed analogous results, but the sex difference was no longer significant after accounting for the specific patient group differences (*F(1,413)* = 2.01, *p* = .16). Bayes factor analyses, assessing the space of models with vs. without main effects and interactions between group and condition, found the most evidence for a model with an effect of condition and diagnosis-specific groups (Bayes factor = 3.68E+10 relative to the null model). It had positive evidence compared to the 2^nd^-best model, which only included trial (Bayes factor = 3.6). Uncorrected post-hoc contrasts suggested the effect of condition reflected greater difficulty in the no-guessing condition relative to the other conditions (*p* < .001 for each) when not including the covariates in the LMEs. The effect of diagnosis-specific group appeared to be explained by lower difficulty in the SUD group than in the HC, ED, DEP+ANX, and DEP groups (*p*s between .001 and .046) when not including the covariates in the LMEs.

*Heart rate***.** For the transdiagnostic (HCs vs all patients) LME analysis, there was a main effect of sex (*F(1,412)* = 5.03, *p* = .03; higher in females), PTT (*F(1,408)* = 6.70, *p* = .01; positive correlation), and trial (*F(1,408)* = 6.70, *p* = .01), and a group by trial interaction (*F(1,408)* = 6.70, *p* = .01). Post-hoc contrasts showed greater heart rate in the breath-hold condition than the other two conditions (*p* < .001 for each), and that the interaction was driven by higher heart rate in patients than HCs in the no-guessing condition (*p* = .02). No other significant main effects or interactions were observed. The diagnosis-specific (HCs vs. 5 separate patient groups) LME analysis showed analogous results, but the group by condition interaction was no longer significant (*F(1,815)* = 1.43, *p* = .14). Bayes factor analyses, assessing the space of models with vs. without main effects and interactions between group and condition, found the most evidence for a model including condition and transdiagnostic grouping (Bayes factor = 3.36E+4 relative to the null model). There was relatively weak evidence favoring this model over a few others, including 1) a model with transdiagnostic group, condition, and their interaction (Bayes factor = 1.4), 2) a model with only an effect of condition (Bayes factor = 1.9), and 3) a model with diagnosis-specific group and condition (Bayes factor = 2.4). Uncorrected post-hoc contrasts suggested the effect of condition was due to greater heart rate in the breath-hold condition than the other two conditions (*p* < .001 for each) and greater heart rate in the guessing than no-guessing condition (*p* = .048), and that the effect of group reflected greater heart rate in patients than HCs (*p* = .035) when not including the covariates in the LMEs. The interaction between transdiagnostic group and condition in the 2^nd^-best model appeared to reflect greater heart rate in the patient group than HCs in the guessing and no-guessing conditions (*p* = .02 and .01, respectively) when not including the covariates in the LMEs. The interaction between diagnosis-specific group and condition in the 3^rd^-best model appeared to reflect greater heart rate in SUDs than HCs (*p* = .01) when not including the covariates in the LMEs.

**Associations with symptom severity**

Given the heterogeneity in our clinical sample, we ran subsequent exploratory correlational analyses with continuous scores on the clinical measures gathered, excluding the healthy comparisons, to assess whether model parameters in the heartbeat tapping conditions might provide additional information about symptom severity. The only notable (but weak) relationships observed were as follows. In the no-guessing condition, IP was negatively associated with both depression (PHQ; *r* = -.13, *p* = .01) and anxiety (OASIS; *r* = -.11, *p* = .03) severity, and positively associated with substance use severity (DAST; *r* = .10, *p* = .04); pHB was also positively associated with substance use severity (DAST; *r* = .17, *p* = .001). In the breath-hold condition, pHB was positively associated with substance use severity (DAST; *r* = .16, *p* = .002). Higher anxiety (OASIS) was also associated with a slightly more anticipatory response pattern (AvR; *r* = -.12, *p* = .02).
