# Supplementary figures and images for: A Bayesian computational model reveals a failure to adapt interoceptive precision estimates across depression, anxiety, eating, and substance use disorders
Source: PLoS Comput Biol. 2020 Dec 14;16(12):e1008484. doi: 10.1371/journal.pcbi.1008484 (PMC7769623; doi:10.1371/journal.pcbi.1008484)

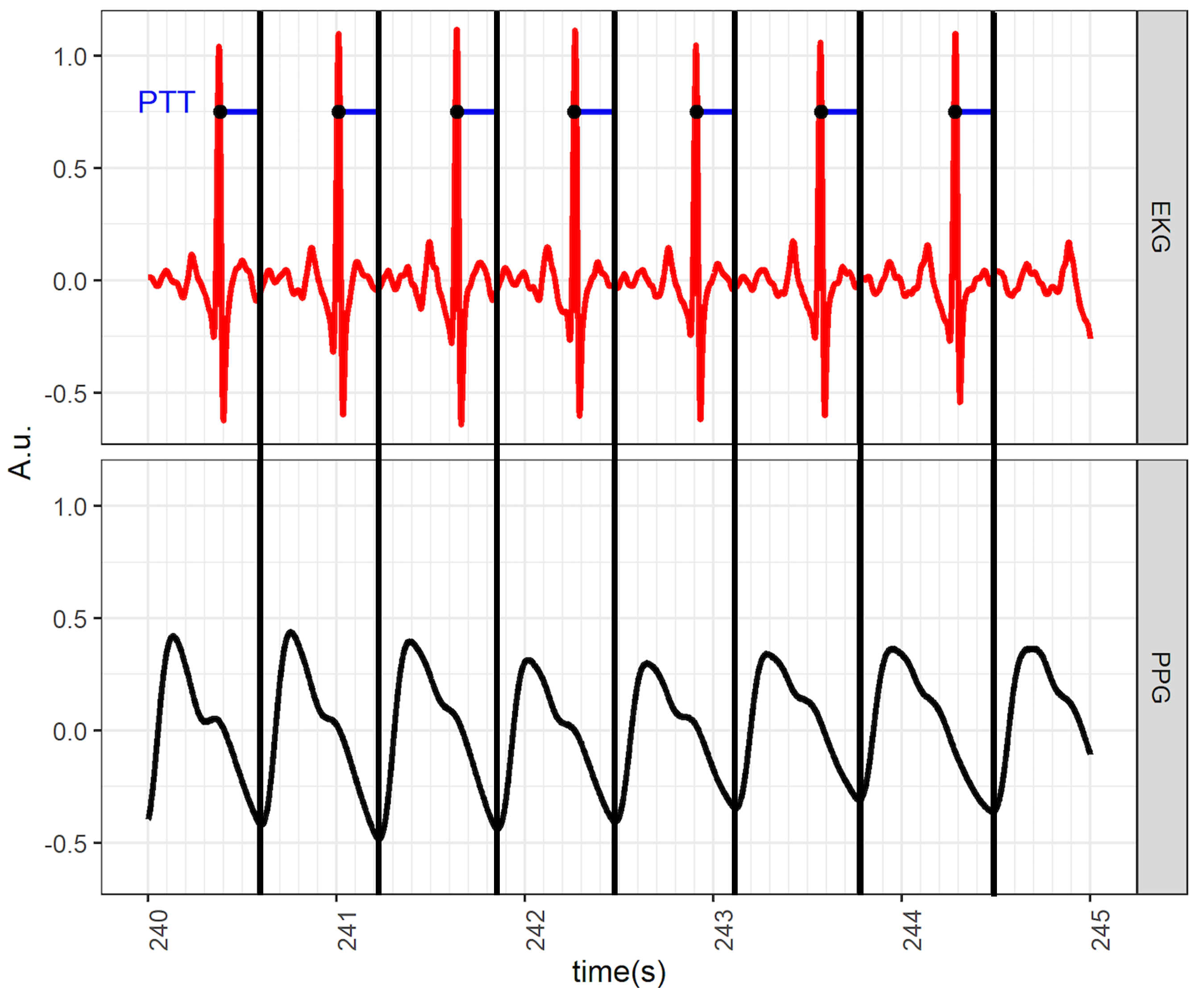

Supplement: S1 Fig — Top: Example EKG trace segment from one participant (red). Bottom: Simultaneous PPG trace from the same participant. Each vertical black line denotes the upswing in the PPG signal after each EKG R-spike. Each cyan line indicates the length of the delay (i.e., the pulse transit time; PTT) between each R-spike and each subsequent upswing in PPG, the median of which was calculated for each participant. These showed that a delay of 200ms was a good estimate of PTT, which is what was assumed for computational modelling. (TIF) [file pcbi.1008484.s002.tif]

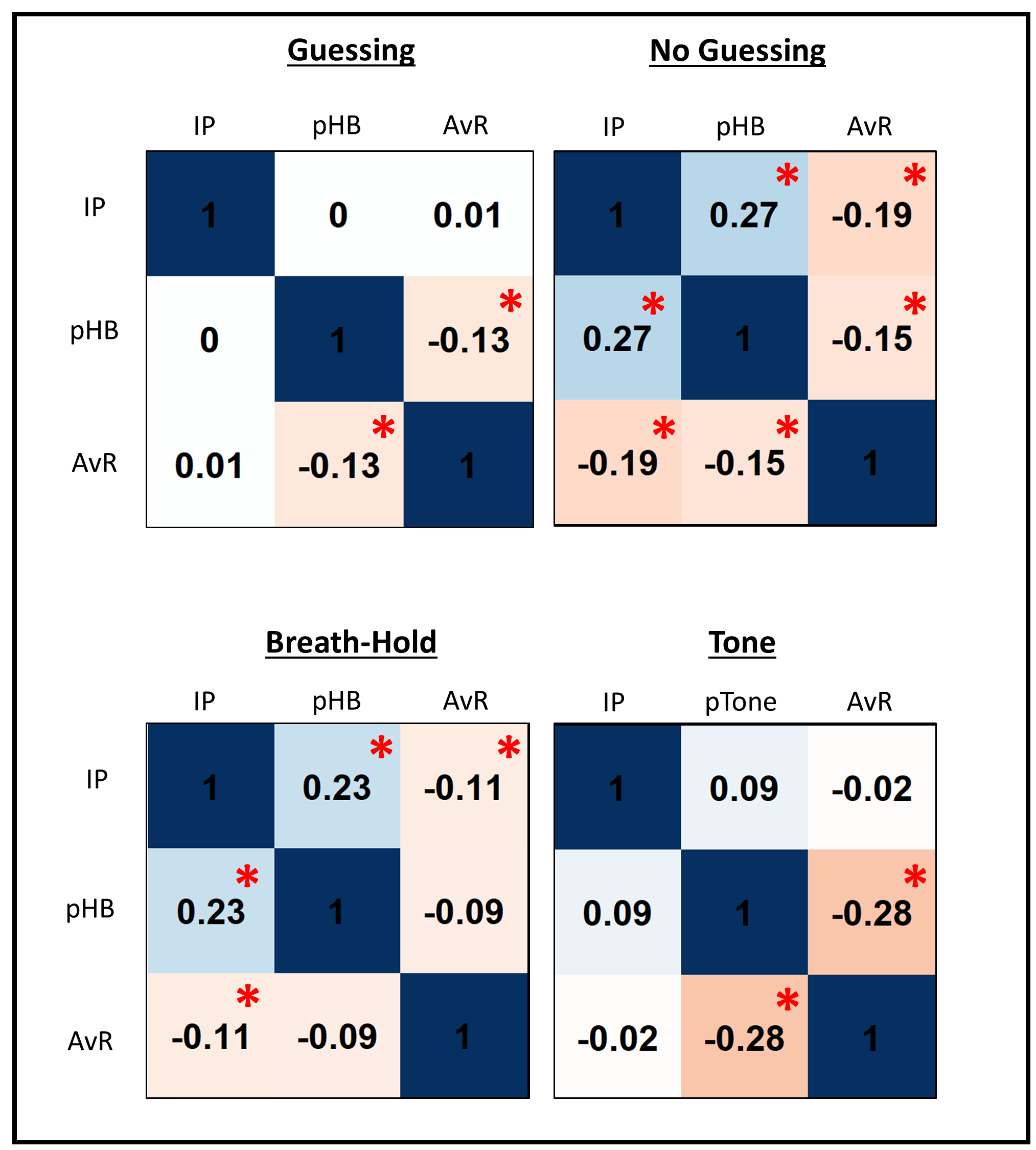

Supplement: S2 Fig — For reference, correlations at p < .05 are marked with red asterisks. (TIF) [file pcbi.1008484.s003.tif]

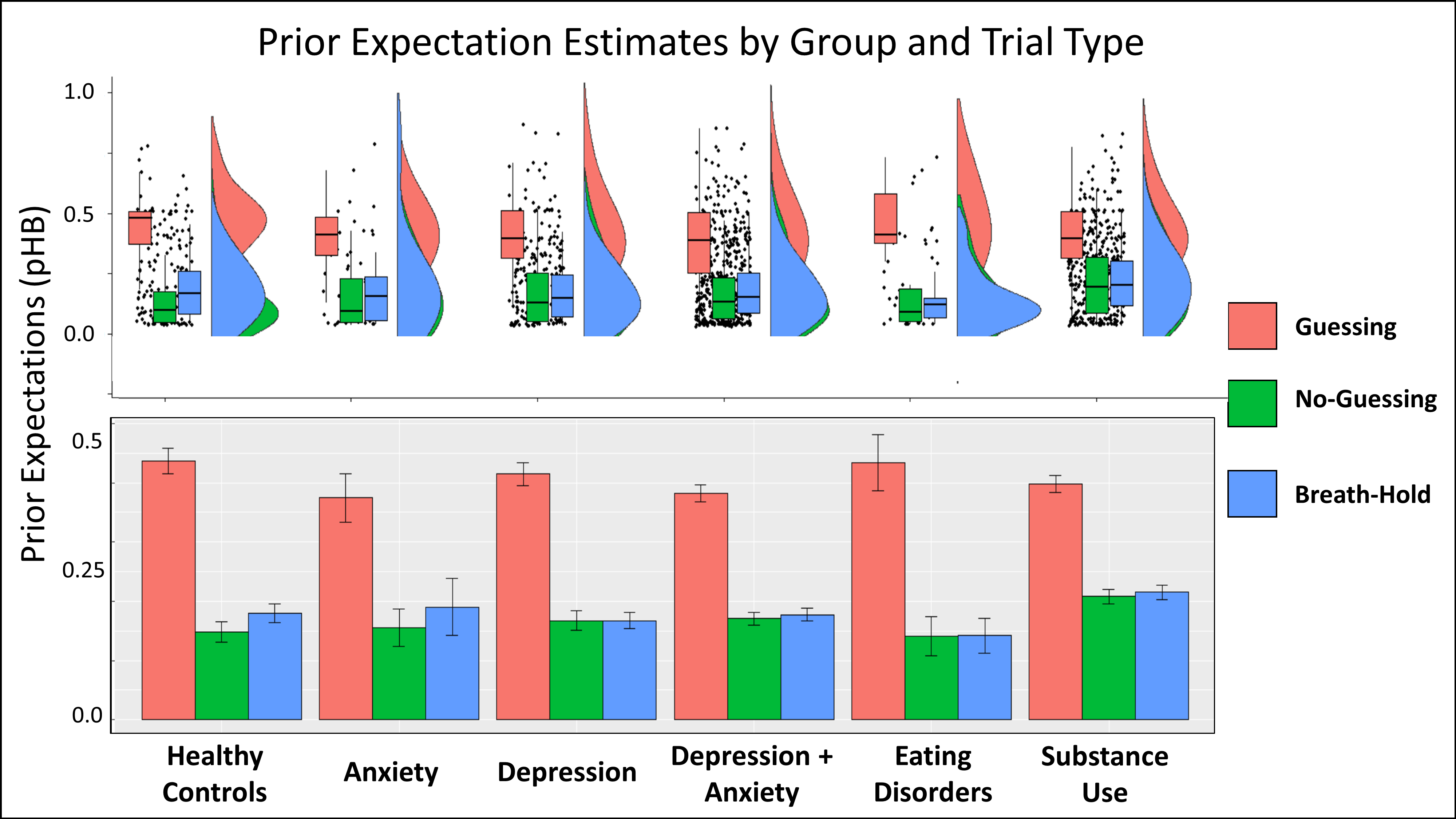

Supplement: S3 Fig — Bottom: Mean and standard error for prior expectation estimates by condition and clinical group. Prior expectations (pHB) were significantly higher in the guessing condition, but were not significantly different between groups in the analyses reported in the main text (although marginally greater pHB in the no-guessing and breath-hold conditions can be seen in the substance use group relative to some of the other groups). Top: Raincloud plots showing the same results in terms of individual datapoints, boxplots, and probability densities. (TIF) [file pcbi.1008484.s004.tif]

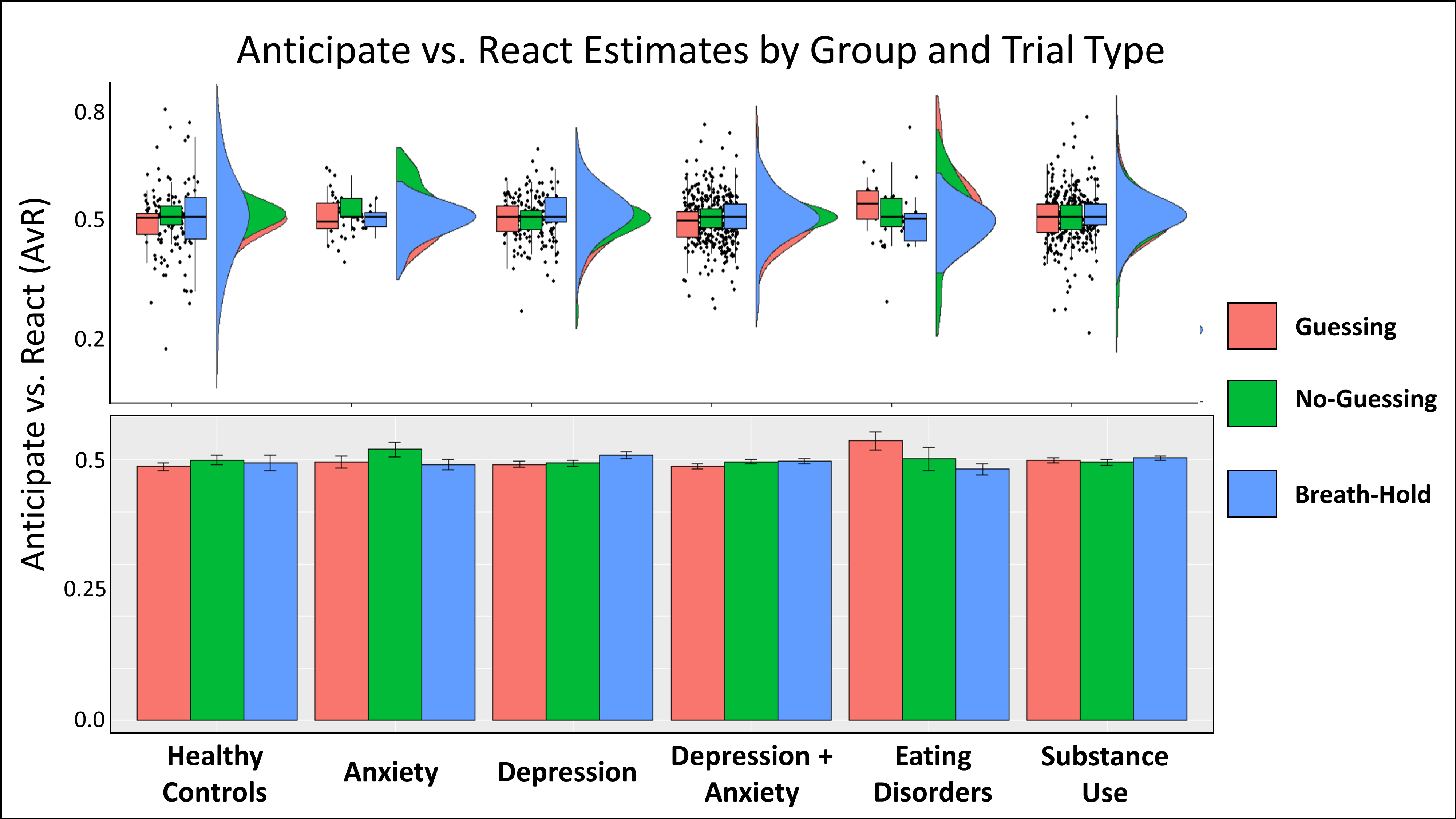

Supplement: S4 Fig — Bottom: Mean and standard error for Anticipate vs. React (AvR) parameter estimates by condition and clinical group. AvR was not significantly different between groups in the analyses reported in the main text. Top: Raincloud plots showing the same results in terms of individual datapoints, boxplots, and probability densities. (TIF) [file pcbi.1008484.s005.tif]

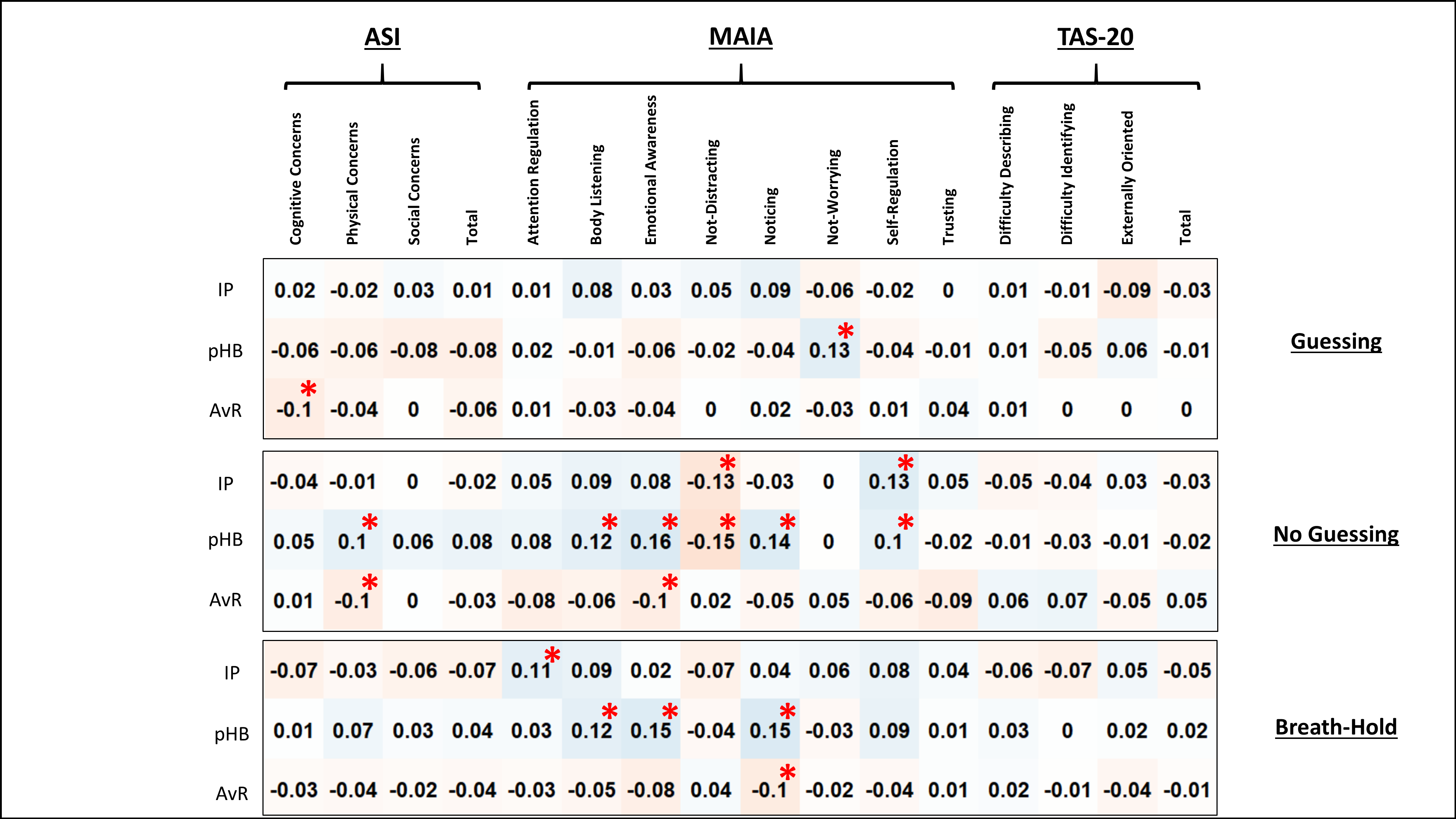

Supplement: S5 Fig — For reference, correlations at p < .05 (uncorrected) are marked with red asterisks. IP = interoceptive precision, pHB = prior expectation for heartbeat, AvR = anticipatory vs. reactive tapping strategy parameter. (TIF) [file pcbi.1008484.s006.tif]
